# The Masquelet Technique for Membrane Induction and the Healing of Ovine Critical Sized Segmental Defects

**DOI:** 10.1371/journal.pone.0114122

**Published:** 2014-12-02

**Authors:** Chris Christou, Rema A. Oliver, Yan Yu, William R. Walsh

**Affiliations:** Surgical and Orthopaedic Research Laboratories, University of New South Wales Australia, Sydney, New South Wales, Australia; Faculté de médecine de Nantes, France

## Abstract

The healing of critical sized segmental defects is an ongoing clinical problem. No method has achieved pre-eminence. The Masquelet technique is a relatively new innovation involving the induction of a fibrous tissue membrane around the bone defect site taking advantage of the body’s foreign body reaction to the presence of a polymethylmethacrylate (PMMA) spacer. The aim of this study was to investigate the properties and characteristics of this induced membrane and its effectiveness when used in conjunction with allograft or an allograft/autograft mix as filler materials in an ovine critical sized defect model. The resultant induced membrane was found to be effective in containing the graft materials in situ. It was demonstrated to be an organised pseudosynovial membrane which expressed bone morphogenic protein 2 (BMP2), transforming growth factor- beta (TGFβ), vascular endothelial growth factor (VEGF), von Willerbrand factor (vWF), interleukin 6 (IL-6) and interleukin 8 (IL-8). While more new bone growth was evident in the test groups compared to the controls animals at 12 weeks, the volumes were not statistically different and no defects were fully bridged. Of the two graft material groups, the allograft/autograft mix was shown to have a more rapid graft resorption rate than the allograft only group. While the Masquelet technique proved effective in producing a membrane to enclose graft materials, its ability to assist in the healing of critical sized segmental defects when compared to empty controls remained inconclusive.

## Introduction

The treatment of critical sized segmental defects is an ongoing clinical problem. The delayed unions and non-unions that can occur as a result of poor patient healing response to stabilisation and grafting techniques currently in use, places extra demand not only on the patient and surgeon, but also on the costs incurred in the hospital system through increased lengths of hospital stays and re-operations [1], [2]. As the incidence of segmental bone loss through trauma increases, for example in motor vehicle accidents or injuries acquired as a result of the many ongoing global conflicts, so does the need to find suitable and effective treatments for these situations. A high proportion of these injuries will be associated with significant amounts of environmental foreign body and bacterial contamination.

Autograft is considered the gold standard void filler for treating bone loss during fracture fixation; it provides osteoconductive, osteoinductive and osteogenic factors at the site of injury [3]. The large volumes of graft material required to fill critical sized segmental defects leads to an increase in the incidence of co-morbidities associated with the harvesting of large amounts of autograft, its exclusive use in these scenarios can often outweigh the benefits. Materials used to help decrease the reliance on autograft are mostly osteoconductive, they can however, be combined with proteins such as bone morphogenic proteins (BMPs) [4], [5] or vascular endothelial growth factor (VEGF) [6], to bestow some osteoinductive properties to these materials as well. No synthetic supplementary graft materials have proven to be osteogenic [7]–[10]. Despite their promise, complications associated with the addition of inductive proteins into graft materials include, the need for supraphysiologic concentrations [4], their potential to cause ossification in adjacent unwanted sites [11], the inability to control their timing of release and some reports which indicate a potential risk of cancer [12]. Containment of graft materials within an open defect site is also a hurdle that needs to be overcome. Synthetic polymer sheets [13], [14] and titanium cages [15] have been used for containment, but they provide no assistance to the healing processes. Blood clots have been proposed and used as a method of forming a mouldable cohesive material for use across defects with the added benefit of providing some healing tissue factors, however mixed results have been achieved [16], [17].

Early this century a surgical technique was described and published by Alain Masquelet [18] which was aimed at helping solve the problems of graft containment and the need for growth protein supplementation of non-autogenous bone grafts. The technique has been well described elsewhere and the details of which will not be entered into here [18]–[23], suffice to say that it is a two-step process which involves the induction of a fibrous membrane prior to the introduction of graft material into a defect site [24], [25]. As a two-step procedure, the first step may not only be used as the setup for membrane induction, but it also provides an opportunity to debride any necrotic tissues and to address and treat any infections prior to the commencement of the healing process at stage two. Many claims have been made regarding the properties and benefits of this induced membrane [19], [23], [26]. The aim of this study was to investigate the induced membrane, its properties, characteristics and its effectiveness in assisting bone healing. This was achieved by applying it to a pre-clinical ovine critical sized defect model developed within our laboratory (in press). An empty control group was compared to defects filled with either allograft or an allograft/autograft mix. Histology, immunohistochemistry and micro computed tomography (microCT) were used as end points.

## Methods

### Allograft preparation

Eight sheep humeri from 5 year old animals which became available from other projects were collected and cortico-cancellous allograft bone was produced following a modified protocol established by Russell et al [27]. Particle sizes in the range of 700 µm–1700 µm were produced [28]–[30]. The density of allograft particles produced was 0.66 g/cm^3^. The volume of the defect to be filled was 16.5 cm^3^ (based on an external tibial shaft diameter of 22 mm and an internal diameter of 8 mm, as reamed during surgery). The resultant particles were divided into 10.9 g and 6.4 g aliquots and sent to Steritech (Sydney, Australia) for gamma sterilisation at 25 kGy on dry ice.

### Surgery

Following ethical approval from the Animal Care and Ethics Committee (ACEC) of the University of New South Wales, approval number 11/126B, 24 adult crossbred ewes (with a mean age of 5.6±0.4 years and a mean weight of 55±5 kg) were commissioned, divided into pairs and acclimatised in their respective stalls (6 m^2^) for one week prior to surgery. They were maintained on a diet of lucerne hay, chaff and water ad lib. The sheep were allocated into 3 groups, an empty control group (Group 1), a 100% allograft group (Group 2) and an allograft/autograft group (67% allograft/33% autograft - Group 3). The control group was taken out to 12 weeks, each of the graft material groups were then divided into 2 time points, n = 4 at 6 weeks and n = 4 at 12 weeks, resulting in one group of 8 animals (control) and 4 groups of four sheep (graft materials).

Sheep were given pre-emptive analgesia 72 hours prior to each surgery using a transdermal fentanyl (100 µg/hr) patch (Durogesic - Janssen, Sydney, Australia). Twelve hours prior to surgery, all food was removed from the stalls. On the day of the surgeries the fentanyl patch was replaced to provide another 72 hours of analgesia post-operatively. The sheep were sedated with an intramuscular (IM) injection of Tiletamine/Zolazepam 5 mg/kg (Zoletil – Virbac Animal Health, Sydney, Australia) prior to gaseous anaesthetic mask induction and maintenance with isoflurane at 2–3% (Abbott, Sydney, Australia). The cephalic vein of the right forelimb was catheterised using an 18 g intravenous (IV) catheter and a 1L bag of 0.9% NaCl was set up at a fluid rate of 1.5× maintenance. Immediately prior to surgery the sheep were pre-medicated with 2 mg/kg carprofen IV (Rimadyl – Pfizer, Sydney, Australia), 10 mg/kg of long acting oxytetracycline IM (Troy Ilium, Sydney, Australia) and 20 mg/kg of cephalexin IV (Virbac Animal Health, Sydney, Australia). For the surgery, the sheep were placed in dorsal recumbancy and a cranio-medial approach to the tibia was used. The midshaft of the left tibia was identified as a point 20 mm from where the ridge of the tibial crest flattens and meets the tibial diaphysis. Measuring 25 mm proximal and distal to this point, a 5 cm mid-tibial osteo-periosteal defect was produced using a saline cooled oscillating saw (Microaire – Zimmer, Australia). The tibia was stabilised using an 8 mm×185 mm stainless steel intramedullary (IM) nail with two proximal and two distal 4.5 mm cross-locking bolts (Innovative Animal Products, Rochester, MN, USA). At the time of nail locking the piece of bone that was removed was split in half longitudinally and placed across the defect to maintain the correct spacing. The eight control animals had their soft tissues closed over an empty defect. For the graft material groups, the defect space was filled with medical grade polymethylmethacrylate (PMMA), Simplex P with tobramycin (Stryker, Sydney, Australia), prior to closure. The amount of PMMA used was individualised per animal. It was moulded such that it replicated the outer cortical dimensions of the tibia and overlapped the ends of the osteotomy site by a few millimetres to prevent the encroachment of soft tissue into the defect space.

**Figure 1:**
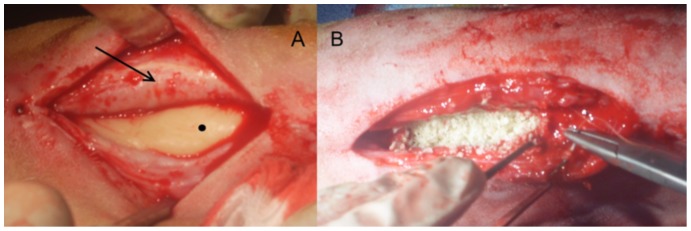
Intra-operative image of PMMA spacer (dot) and capsule (arrow) (A); Suturing the capsule closed after filling with graft material (B).

Four weeks after the first surgery, the sheep of groups 2 and 3 were re-anaesthetised using the same anaesthetic and analgesic protocol as described above. The surgical site of each sheep was approached via a direct incision over the original wound, the subcuticular tissues were incised in the same plane leading directly to the underlying bone cement across the defect. The fibrous capsular tissue surrounding the PMMA spacer was identified and a small sample taken for histological and immunohistochemical evaluation (Figure 1A). The spacer was then drilled and chiselled out with care so as not to damage the capsule. Following removal of the spacer, the osteotomy ends of the bone were curetted so as to simulate a recent fracture in an effort to stimulate bone growth following the 4 week suppression caused by the presence of the PMMA. The fibrous capsule pocket/defect was evenly filled with the graft material for each of the two groups respectively and the capsule was closed as a separate layer to the remaining soft tissues of the area (Figure 1B). Cancellous bone was harvested from the greater trochanter of the left humerus using a 6 mm drill bit and curette for use in the allograft/autograft group (Gp3).

### Imaging

Post-operative x-rays were taken immediately after each surgery using a portable X-ray machine (Poskom, Korea) and digital plates (Agfa, Scoresby VIC, Australia.) at 65 kV and 50 mAs.

At the time of harvest for each time point, the nails were carefully removed avoiding disruption of the tissues within the defect allowing for artefact free micro computed tomography (microCT) scans (Inveon Siemens, Erlangen, Germany). Four bed microCT scans were performed to encompass the whole tibia using 80 kV/500 µA, with a resolution (pixel size) of 54 µm. The data was imported into Mimics software version 15 (Materialise, Leuven, Belgium) for image analysis. All the images were thresholded to remove any soft tissue and cartilage, leaving only trabecular and cortical bone. The thresholding was performed visually based on the removal of the remaining soft tissue present on the tibia during the scanning which remained evident on the image. Three dimensional (3D) images were constructed and then sectioned at the level of the osteotomy to isolate the defect site. This resulted in a region of interest (ROI) encompassing the whole defect. Analysis of the volume of new bone growth and residual graft material was performed using SPSS (IBM SPSS Statistics 20) based on these images and the results compared across the groups with independent sample t-tests with a p-value set at 0.05. Animals 2376 and 2389 were excluded for the residual graft material statistical analysis due to the presence of infection.

### Histology - Capsule

The harvested capsular tissue (collected when removing the spacer) was preserved in 10% phosphate buffered formalin solution. The tissues were processed for paraffin histology by dehydrating through a graded alcohol series and cleared in xylene before embedding in paraffin wax. After trimming, 5 µm serial sections were cut from each block using a Leica Microtome (Leica MicroSystems GmbH) and mounted onto silanized slides for histological and immunohistochemical examination.

Slides were stained with Harris’ hematoxylin and eosin (H & E), for tissue and cellular identification. Cells were identified based on stain uptake and morphology across 5 high power fields (400×) for each slide. Further differentiation of cell type was performed under oil immersion (1000×).

### Histology – Defect

After imaging, the defects were isolated from the tibial diaphysis and preserved in 10% phosphate buffered formalin solution, prior to decalcification in a 10% formic acid-phosphate buffered formalin solution to be processed for histology. Once fixed and decalcified the defects were cut through the middle in 2 planes, one parallel to the long axis of the tibia, producing coronal sections and the other perpendicular to it, resulting in proximal and distal sections. This provided four sample areas per defect, proximal cranial, proximal caudal, distal cranial and distal caudal. Histological processing followed the same protocol as that used for the capsular tissue, with the addition of Tetrachrome staining.

### Immunohistochemistry - Capsule

A standard immunohistochemical procedure was performed on all 5 µm paraffin sections of the harvested capsular tissue for the expression of; bone morphogenic protein (BMP2), vascular endothelial growth factor (VEGF), von Willerdrand factor (vWF), transforming growth factor beta (TGF-β), interleukin-6 (IL6) and interleukin-8 (IL8) [31]. After deparaffinisation, a heat based antigen recovery was performed on the tissues using a citrate based solution (DAKO Pty Ltd, Glostrup, Denmark) at 100°C for 30 minutes. Endogenous peroxidase was quenched in a 0.3% hydrogen peroxide in a 50% methanol solution for 10 minutes followed by a rinse in phosphate buffered saline (PBS) with 0.01% tween-20. Primary mouse monoclonal antibodies (Santa Cruz Biotechnology, CA, USA) against BMP2 2 µg/ml, VEGF 5 µg/ml, vWF 1∶1000, IL-6 0.2 mg/ml and IL-8 2 µg/ml, were used along with non-immunised mouse immunoglobulin (mouse IgG) (DAKO Pty Ltd, Glostrup, Denmark) 5 µg/ml as a negative control. Primary rabbit monoclonal antibodies against TGF-β 4 µg/ml were used along with non-immunised rabbit immunoglobulin (rabbit IgG) (DAKO Pty Ltd, Glostrup, Denmark) 4 µg/ml as a negative control. The slides were left overnight at 4°C, washed with PBS/tween-20 and incubated with Envision System-HRP Labelled Polymer (anti-mouse K4001, DAKO Cytomation, CA) for one hour. After washing with PBS/tween-20, a substrate-chromogen system, DAKO liquid diaminobenzidine (K24668, DAKO Pty Ltd, Glostrup, Denmark) was applied for 30 minutes. The reaction was stopped by rinsing in PBS, counterstained using Harris hematoxylin, and mounted with coverslips. Qualitative analysis was performed in a blinded fashion by the authors (CC and RO) across five fields (200×) per slide under light microscopy. For the capsular tissue a grading scale of negative (−ve) to three plus (3+) was used to indicate the level staining for intensity, quantity and distribution of protein expression.

### Immunohistochemistry – Defect

Following the above mentioned protocol, 5 µm paraffin sections of the 12 week defect tissues were also examined for the expression of BMP2, VEGF, TGF-β, vWF and IL-6 with additional staining for cathepsin K (CTSK) and alkaline phosphatase (ALP) included. Primary mouse monoclonal antibodies against CTSK 0.1 µg/ml (Santa Cruz Biotechnology, CA, USA) and ALP 0.5 µg/ml were used. The defect tissues were qualitatively assessed for factor expression distribution.

## Results

### Surgery

All the surgeries went well and there were no complications. Post-operative management followed the same protocol as previously published (in press).

At 4 weeks post-operatively a draining sinus was seen in 2 of the graft group animals. One was from the allograft/autograft group and the other from the autograft only group. Both were treated with penicillin for two weeks which improved the symptoms and the sinuses resolved. These two animals were allocated to the 6 week time point.

### Imaging

**Figure 2:**
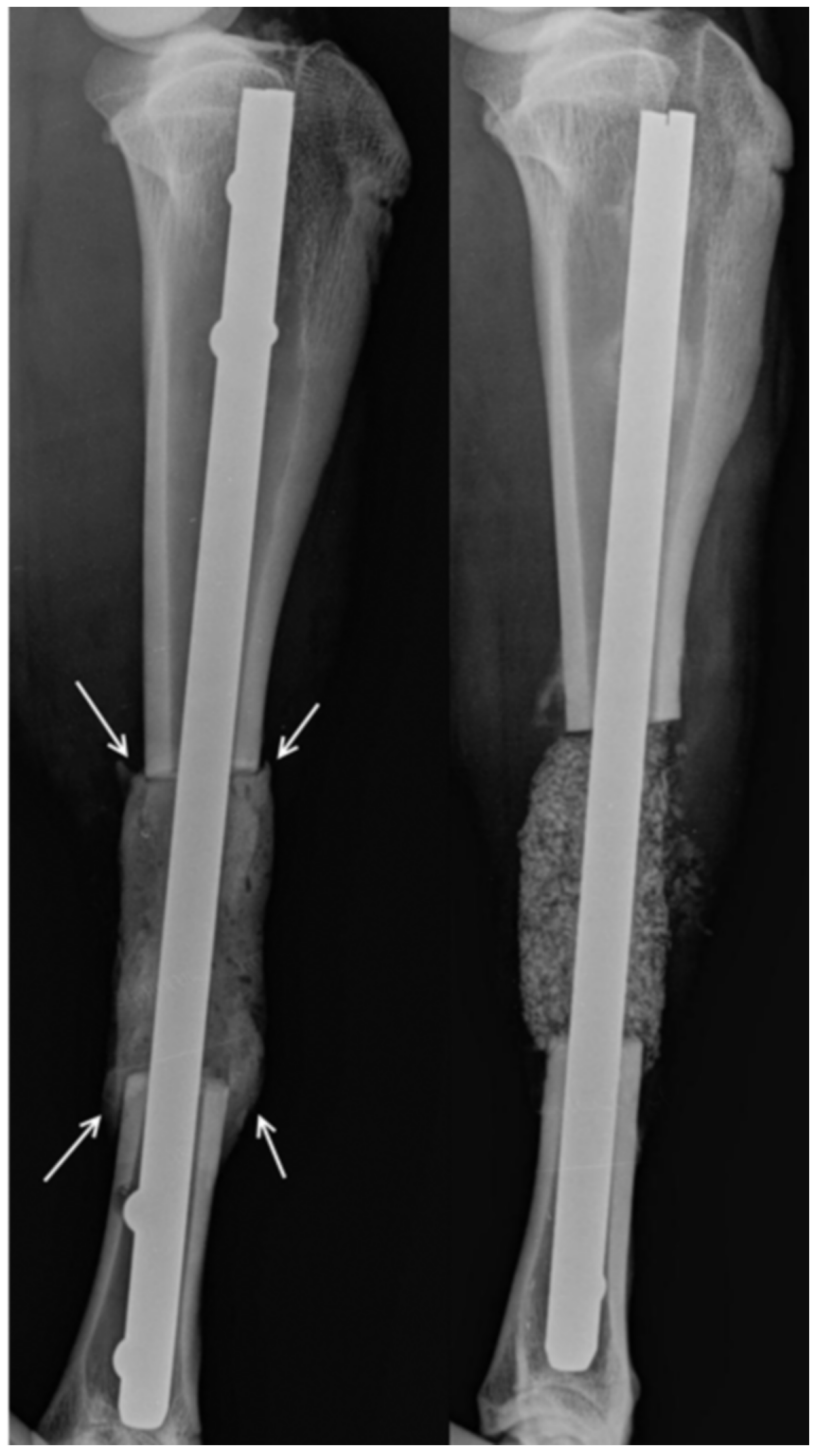
Post-operative radiographs showing PMMA spacer with overlapping edges after the first surgery on the left and the graft filled capsule following the second surgery on the right.

Post-operative x-rays showed good overlap of the PMMA added across the defect at the first surgery and a capsule full of graft material at the second surgery (Figure 2).

**Figure 3:**
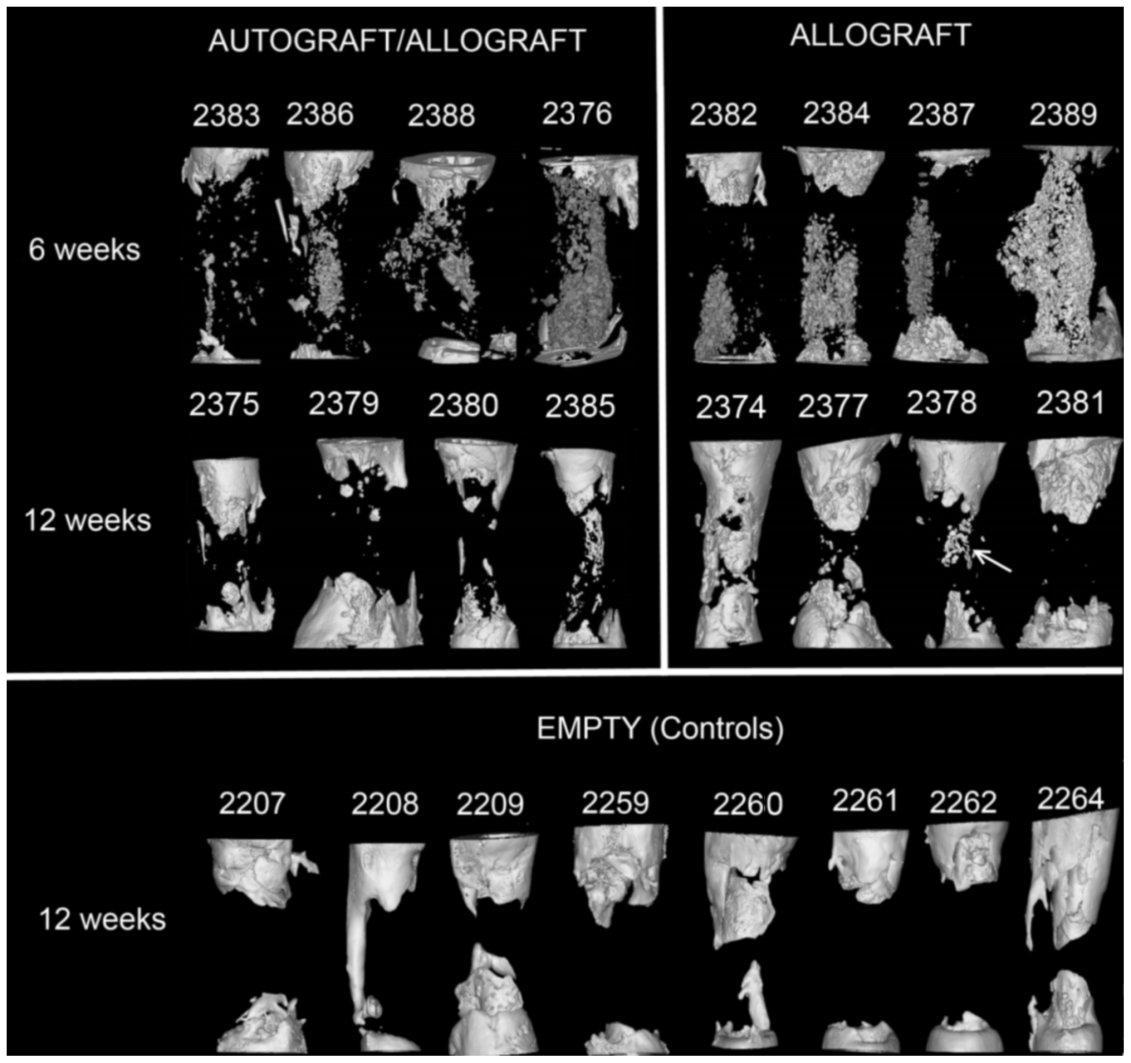
MicroCT scans of the defects from the different groups showing residual graft material at 6 weeks (top row), new bone growth at 12 weeks for graft material groups (middle row) and new bone growth at 12 weeks for the empty group (bottom row). Arrow at 2387 indicates some residual allograft material not yet incorporated into new bone.

At the first time point of six weeks after the removal of the spacer and implantation of the graft material, new bone growth was evident at the osteotomy ends. The residual volume of graft material present in the defect was significantly different between the two groups p<0.05 (Figure 3 top row). A mean residual volume of 323±69 mm^3^ was present in the allograft group and 119±70 mm^3^ for the autograft/allograft group. By the 12 week time point there was no residual graft material present (Figure 3 middle row), having all been resorbed or incorporated into the new bone growth attempting to bridge the defect. The amount of new bone growth between the two graft material groups by the 12 week time point was not statistically significant (p = 0.355), though there was a trend towards more new bone in the allograft only group. When compared to the empty defects (Figure 3 bottom row), the allograft only group had a p value of 0.287 and the allograft/allograft group had a p value of 0.78. All raw data is presented in Table S1 and Table S2.

### Histology - Capsule

**Figure 4:**
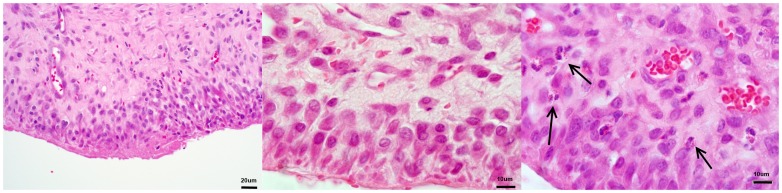
H & E sections of the capsule formed as a result of the PMMA spacer. A: Showing transition of cell orientation deeper into the tissue (400×). B: Showing no basement membrane (1000×). C: Presence of eosinophils (arrows) within the tissue (1000×).

The 4 week old fibrous membrane that formed in the presence of the PMMA spacer consisted of a 3–4 cell layer of densely packed, cuboidal in appearance, fibroblastic type cells adjacent and aligned parallel to the cement spacer. The deeper layers displayed the more commonly encountered elongated fibroblast. Aligned initially parallel to the cement spacer, the fibroblasts slowly became more random in their orientation as distance from the spacer increased, the fibroblasts then blended with the deeper tissues making thickness measurement difficult (Figure 4A). No basement membrane was evident between the surface cells and the deeper cells (Figure 4B). The tissue contained many blood vessels and the occasional polymorphonuclear cell such as a neutrophil or eosinophil (Figure 4C). These cells were mainly found around blood vessels or very close to the surface of the capsule.

### Histology – Defect

**Figure 5:**
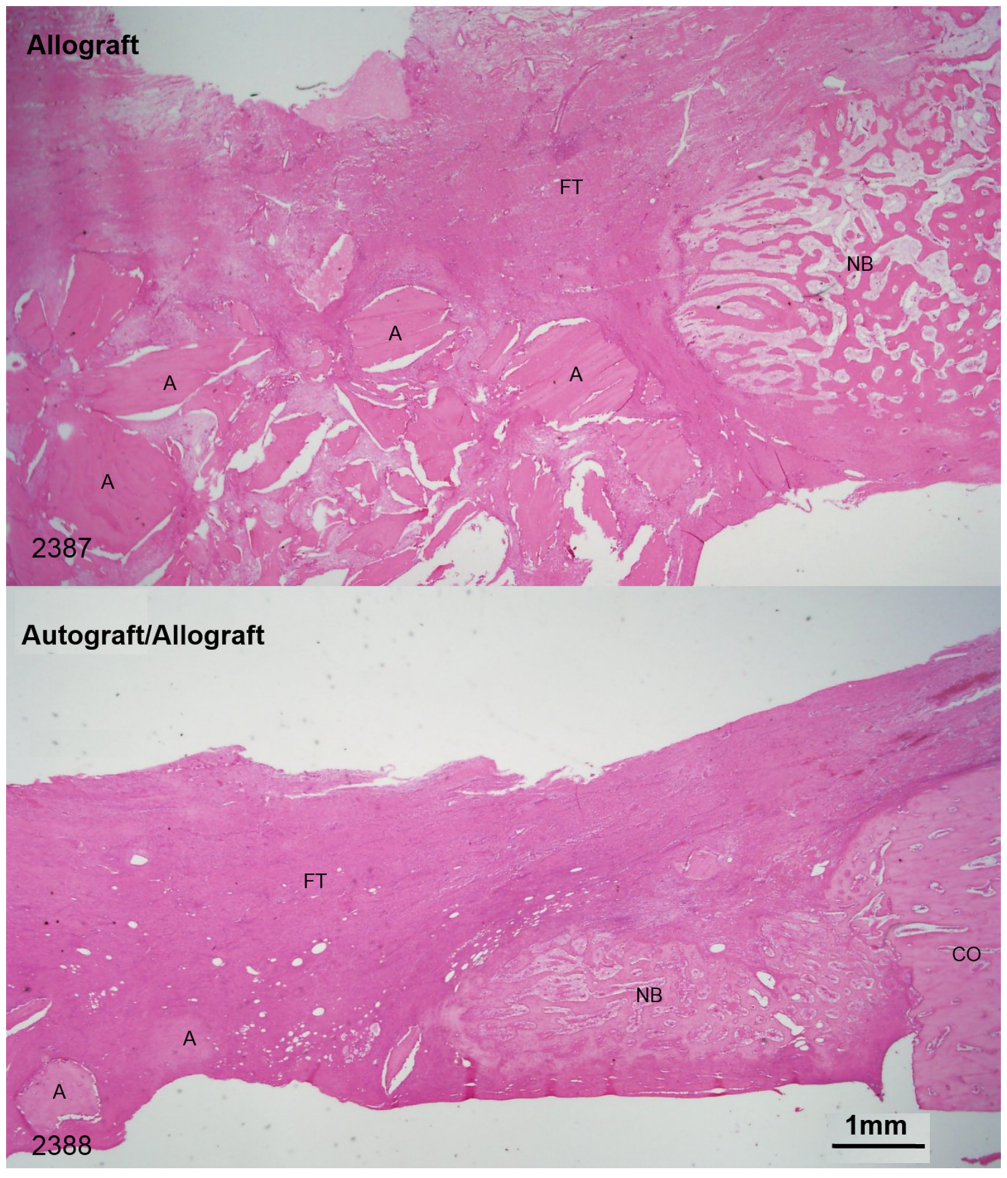
Representative sections of the advancing edge of the callus within the defect at 6 weeks showing fewer remaining graft particles (A) in the allograft/autograft group than the allograft only group (NB = New bone; CO = cortex; FT = Fibrous tissue) (12.5×).

**Figure 6:**
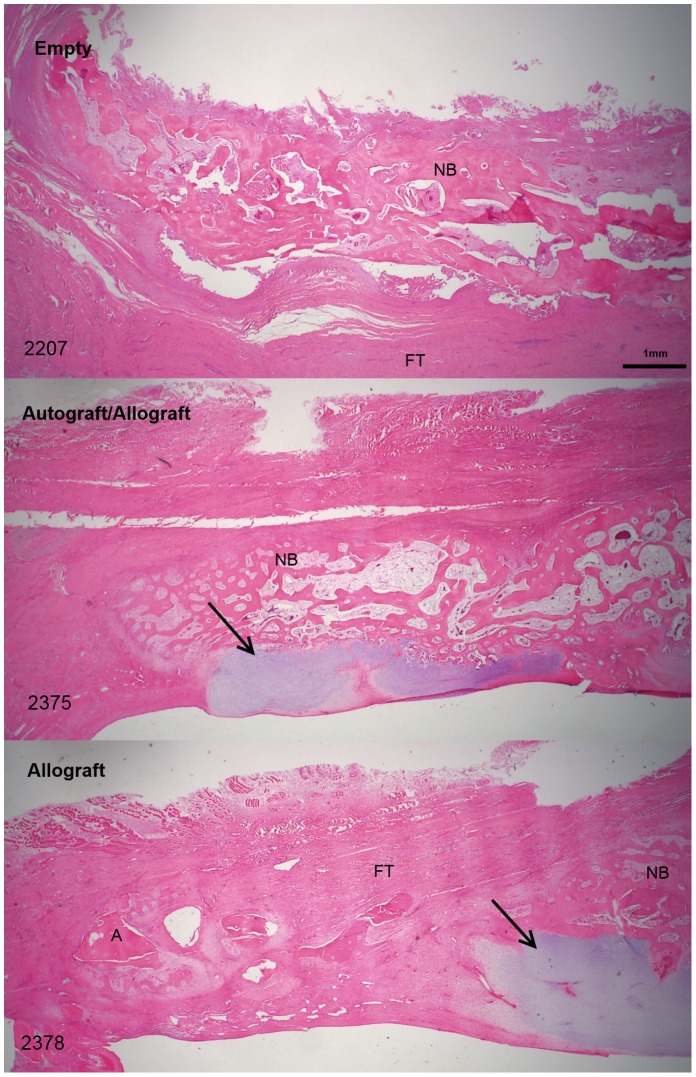
All three 12 week groups where cartilaginous tissues (arrows) can be seen in the two grafted groups as compared to the empty group (12.5×).

Histological sections of the graft material groups at the 6 week time point confirmed the findings presented by the microCT scans, which indicated a more rapid absorption of the autograft material as compared to the allograft material within the autograft/allograft mix (Figure 5 H & E and Figure S1 Tetrachrome). Both H & E and Tetrachrome showed that the induced membrane was no longer distinguishable from the surrounding fibrous tissue that had formed across the defect by the 6 week time point (Figure S2 6 week H & E). At the 12 week time point all animals showed new bone formation across the defect, including the presence of cartilaginous tissues within the grafted groups with little evidence of residual graft material (Figure 6 and S3 12 week composite sections).

### Immunohistochemistry - Capsule

**Figure 7:**
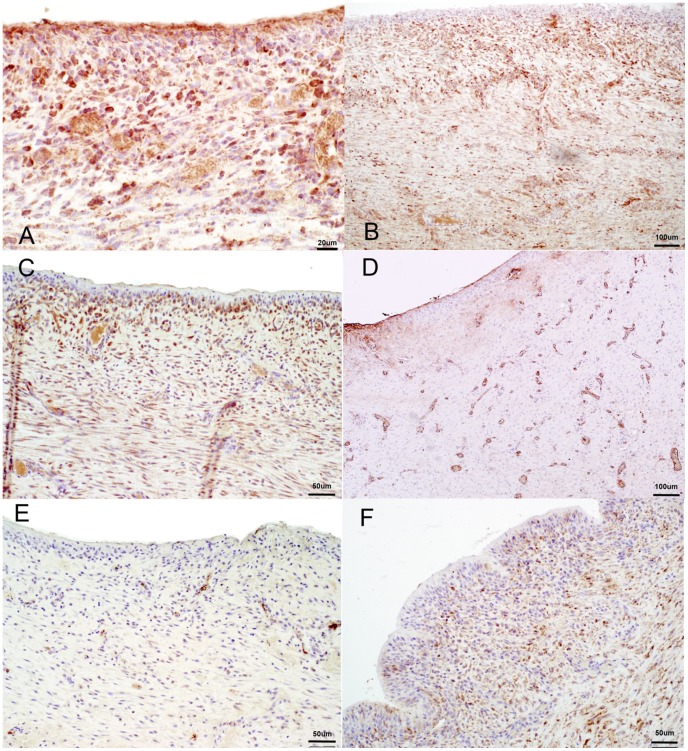
Sections showing immunochistochemical expression. A: BMP2; B: TGBβ; C: VEGF; D: vWF; E: IL-8; F: IL-6.

Each of the factors investigated in the induced membrane stained positive to varying degrees (Figure 7). Their patterns of expression including distribution and intensity of staining are summarised in Table 1.

**Table 1:** Immunohistochemical marker expression, degree and distribution.

Immunohistochemistry factors						
	Role	Staining Results				
		Diffuse	Sporadic	Strong	Regular	Irregular
Bone morphogenic protein 2 (BMP2)	Anabolic	3+	−ve	3+	2+	−ve
Vascular endothelial growth factor (VEGF)	Angiogenisis	1+	1+	1+	−ve	2+
von Willerbrand Factor (vWF)	Angiogenisis	−ve	−ve	2+	1+	−ve
Transforming growth factor beta (TGFβ)	Multiple roles	2+	−ve	2+	1+	−ve
Interleukin 6 (IL-6)	Inflammatory	2+	−ve	2+	1+	−ve
Interleukin 8 (IL-8)	Inflammatory	1+	−ve	−ve	−ve	1+

Qualitative scale of negative (−ve) to 3+.

BMP2 expression (figure 7A) was diffuse, strong, easily isolated and distinctly defined in the cytoplasm, surrounding the nucleus. It was especially visible in the surface cells of the capsule.

TGF-β expression (Figure 7B) was strong, diffuse and evenly distributed, its presence seen in both surface and deeper tissues.

Von Willebrand factor (Figure 7D) stained strongly and specifically, highlighting the blood vessels present in the capsular tissue. In contrast to vWF, VEGF (Figure 7C) was not only evident around the blood vessels, but was more diffusely distributed throughout the capsular tissue. It was, more importantly, present in the surface cells as well.

IL-8 expression (Figure 7E) was found predominantly in the deeper tissues, the staining in general was weak and sporadically distributed. Little or no staining was seen at the 3–4 cell surface of the capsule. IL-6 expression (Figure 7F) was stronger, more diffuse and present in the surface cells as well.

### Immunohistochemistry - Defect

Immunohistochemical analysis of the 12 week defects showed some variable anabolic factor expression (BMP2, TGF-β and VEGF) on the surface of the new bone growth and the surrounding fibrous tissues across the defect (Figures S4 ALP expression, S5 BMP2 expression, S6 TGF-β expression and S7 VEGF expression). ALP expression was consistently strong in all animals and visible on the surface of the newly formed bone without diffusion into the surrounding fibrous tissues (Figure S4). The catabolic factor expression (CTSK) was associated with the allograft material within the defect as well as the deeper surrounding fibrous tissues (Figure S8 CTSK expression). Some evidence of CTSK was also evident close to the osteotomy site (oldest part of the callus (Figure S8)). There was no expression of IL-6 by the 12 week time point in either the empty or grafted groups.

## Discussion

This study evaluated the quality of the fibrous membrane induced by a PMMA spacer across a critical sized segmental defect and its effect on the healing process. Endpoints included histology, immunohistochemistry and radiology.

The fibrous membrane that formed as a result of the body’s foreign body reaction to the presence of the PMMA spacer after 4 weeks was a visibly well-defined tissue. It proved to be an effective graft material containment membrane, across what was initially an open defect site. Histologically it was a tissue stratified in its organisation, with cells orientated relative to the interface between the tissue and the PMMA forming a pseudosynovial membrane [24]. Deeper into the membrane away from this interface the fibroblastic cells became more random in their orientation. Fibroblast cells are of mesenchymal tissue in origin and play an important role in the laying down of extracellular matrix and tissue healing [32]. Immunohistochemical staining revealed a healthy active membrane secreting various tissue factors which have the potential to assist bone healing.

BMP2 staining was diffuse, strong, easily isolated and distinctly defined in the cytoplasm, surrounding the nucleus. It was especially visible in the surface cells of the capsule. Its role in fracture healing and osteogenesis has been well documented [11], [33]-[35], its presence within the induced membrane being beneficial. VEGF is crucial for angiogenesis [36], its ancillary roles will vary according to the prevailing tissue type in which it is found, such as bone repair [37] or assisting follicular growth and development in the ovary [38]. In the present situation its staining pattern appears to indicate a role of providing a new blood supply to the growing and healing tissues. Its osteogenic role however, should not be ignored [39]. Closely associated with VEGF in its staining distribution was vWF, it has been implicated in the regulation and normal formation of blood vessels, its absence leading to angiodysplasia [40], its presence here can only be positive in assisting new tissue growth. The staining intensity and distribution of TGF-β is in keeping with its role in the healing process. As discussed by Sporn [41], the role that TGF-β plays in tissues, is determined by its context and surrounding cytokines. The aforementioned presence and distribution of VEGF, BMP2, and vWF more likely indicates an anabolic, stimulatory role for TBF-β than a catabolic one within this tissue type. IL-6′s staining intensity and distribution is indicative of its dual function of acting as an acute inflammatory stimulator, then becoming a chronic inflammatory modulator and tissue factor stimulator [42]. It can also play a role in osteogenesis and its presence may not necessarily be a negative one in fracture healing [43]. IL-8 proved to have a weak and sporadic distribution in the deeper tissues with no presence in the surface cells. This reflects a waning inflammatory response, indicative of the four week time point, as IL-8 is an acute inflammatory cytokine [44].

The residual graft volume results from the microCT imaging showed a faster resorption of the autograft material than the allograft material. This leads to the observation that despite the discovery of growth factor protein production in the induced membrane, the presence of the inflammatory factors IL-6 and IL-8 cannot be ignored and may be counterproductive to the survival of the autograft material. This is in contrast to the autograft sparing effect of the induced membrane as suggested by Masquelet [19], [45]. Further to these observations, the two defects that developed a secondary infection had greater residual graft material than the other defects, without a concomitant decrease in new bone formation across the defect site. It appears here that the inflammatory processes within the defect were otherwise occupied by the bacterial presence. This inflammatory system pre-occupation along with the presence of pus appears to have produced a graft sparing environment (Figure 3–2376, 2389).

While not statistically significant a difference in the amount of new bone growth across the defect was seen between the two graft groups, the slower resorption rate of the allograft than the autograft suggests that the use of an induced membrane to assist in graft containment and provide growth factors at the graft site may benefit from the use of materials which are more resistant to the low grade foreign body inflammatory reaction that exists within this environment.

The combination of intramembranous and endochondral ossification, as evident in the histological sections, indicate a more progressive healing response with further potential for new bone growth in the graft groups than the empty groups, where no cartilaginous tissues were evident and hence intramembranous ossification was the only healing process occurring (Figure 6). The histological findings were backed up by the immunohistochemical findings, which showed a greater presence of the anabolic growth factors, BMP2, VEGF and TGF-β in the grafted groups as compared to the empty group. All of the factors not only stained with an increased intensity at the same concentration levels, but their distribution within the callus was more consistent and wide spread. The presence of CTSK close to the osteotomy site indicates a remodelling process occurring in the more mature areas of the callus indicative of the 12 week time point. The absence of IL-6 by the 12 week time point is also indicative of the subsidence of ongoing inflammation once the foreign material of the PMMA spacer was removed, making this result consistent with the empty group. The presence of the actively induced membrane appears to have helped in raising the concentrations and distributions of the growth factors necessary for healing. This was reflected in more new bone growth in the grafted groups, however the difference was not statistically significant.

While sixteen sheep were used in total for this study, providing a good sample size for investigation of the induced membrane and established that a non-union was evident by 12 weeks; division of this number into smaller groups for the graft material investigations limited the power of the bone growth comparisons.

Fracture healing can occur over an extended period and this study has established that the induced membrane produces some growth factors at the sampled 4 week time point. Despite the identification of these growth factors, yet to be investigated is how far these factors diffuse into the defect site and what their duration of activity is. This being the case, with current knowledge, IM nailing, by its physical presence within the defect reduces the volume and thickness of the graft material required when compared to plating or external fixation. Logic would therefore suggest that currently, IM nailing should be considered a superior form of fracture fixation when combined with the Masquelet technique. The 12 week histological sections presented in this study showed no obvious remanent of the induced membrane that was distinguishable from the fibrous tissues which eventually bridged the defect. Jin et al. have studied the properties of membranes induced in a subcutaneous environment over a series of time points up to eight weeks and found no significant osteoinductive capacity of the membranes in this environment [46]. Gruber et al. have taken this time point out to 16 weeks in a rat model, however and demonstrated a milieu of genomic factor expression within the membrane, including many which could be detrimental to the presence of autograft such as Cathepsin-K [47].

Looking forward, studies to investigate if PMMA is the ideal material for membrane induction should be considered. Also, are the ideal surface properties of the inductive material to be used smooth or rough and if rough what is the scale of this roughness [48].

## Conclusion

The use of the Masquelet technique shows potential as a method of assisting new bone formation across large defects. The induced membrane proved effective in containing the graft materials, as well as expressing various growth factors. However further investigation is required to find the ideal graft material for use within this procedure.

## Supporting Information

Figure S1 **12 week sections.** Tetrachrome staining of same sections presented in figure 5 in the main manuscript showing greater tissue differentiation than possible with H & E only.
(TIF)

Figure S2 **6 week section.** A representative sample of H & E and tetrachrome staining of a defect of (animal number 2386), showing the integration of the induced fibrous tissue membrane into the surrounding connective tissue by the 6 week time point. The bottom row shows the fibrous tissue orientation adjacent the stainless steel nail being slightly more directional than that of the top row where the fibrous tissue randomly blends with the surrounding tissues at the outer margin of the callus.
(TIF)

Figure S3 **12 week composite sections.** Representative composite H & E slides of the three different groups within the study showing the amount of new bone growth from one side of the defect.
(TIF)

Figure S4 **ALP expression.** Representative slides of ALP staining from each of the defect groups
(TIF)

Figure S5 **BMP2 expression.** Representative slides of BMP2 staining from each of the defect groups
(TIF)

Figure S6 **TGF-β.** Representative slides of TGF-β staining from each of the defect groups
(TIF)

Figure S7 **VEGF.** Representative slides of VEGF staining from each of the defect groups
(TIF)

Figure S8 **CTSK.** Representative slides of CTSK staining showing activity near the osteotomy site (as indicated by the black line) top, and surrounding the graft material, below.
(TIF)

Table S1 **Bone volume data.** Raw bone volume data calculated in Mimics from the microCT scans. Figures presented are the volumes of new bone growth across the sectioned region of interest as presented in Figure 3 of the main manuscript at the 12 week time point.
(XLSX)

Table S2 **Graft volume data.** Raw graft volume data calculated in Mimics from the microCT scans. The figures presented are the residual bone graft volumes at the 6 week time point.
(XLSX)
