# Supplementary figures and images for: The Masquelet Technique for Membrane Induction and the Healing of Ovine Critical Sized Segmental Defects
Source: PLoS One. 2014 Dec 2;9(12):e114122. doi: 10.1371/journal.pone.0114122 (PMC4252083; doi:10.1371/journal.pone.0114122)

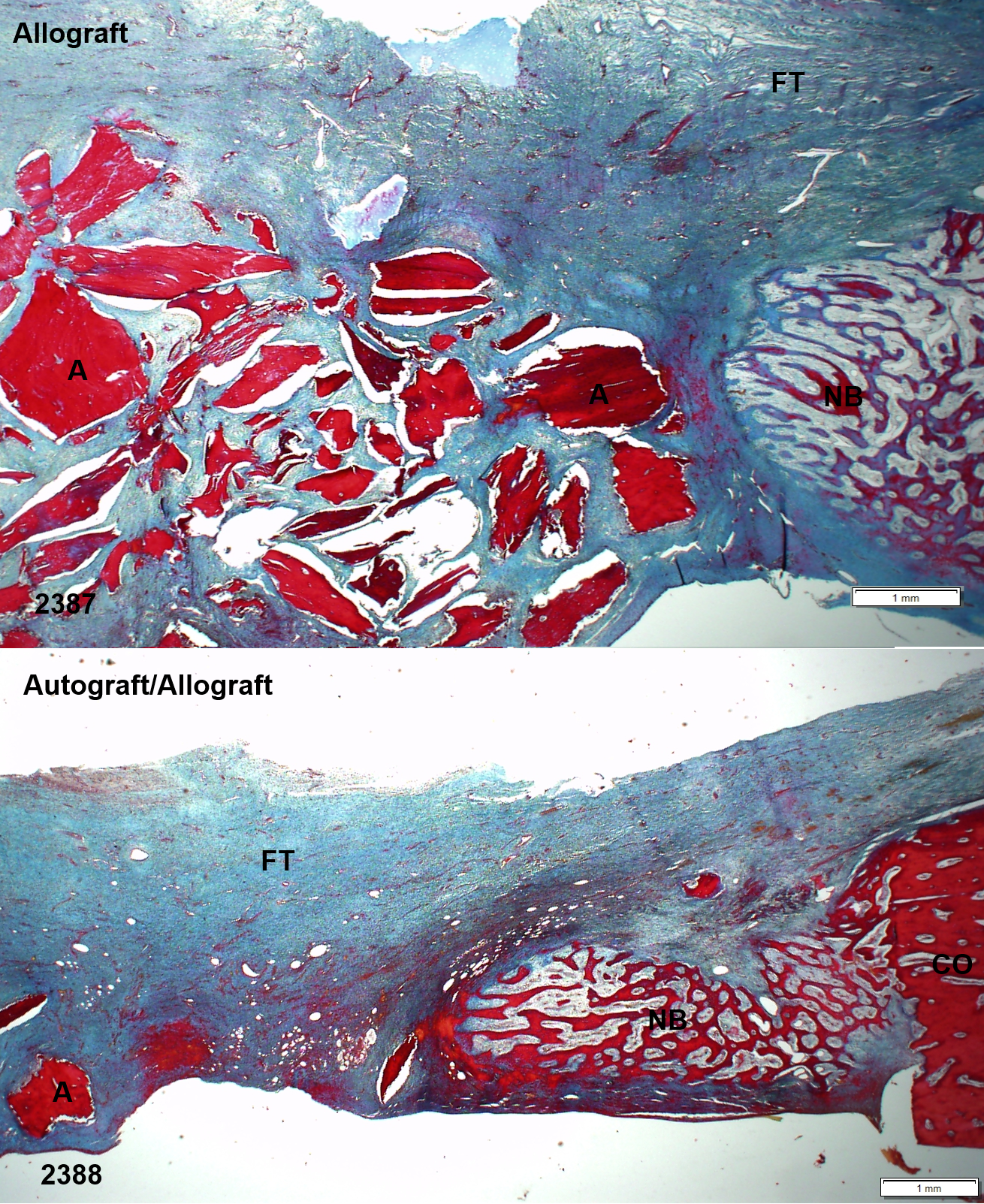

Supplement: Figure S1 — 12 week sections. Tetrachrome staining of same sections presented in figure 5 in the main manuscript showing greater tissue differentiation than possible with H & E only. (TIF) [file pone.0114122.s001.tif]

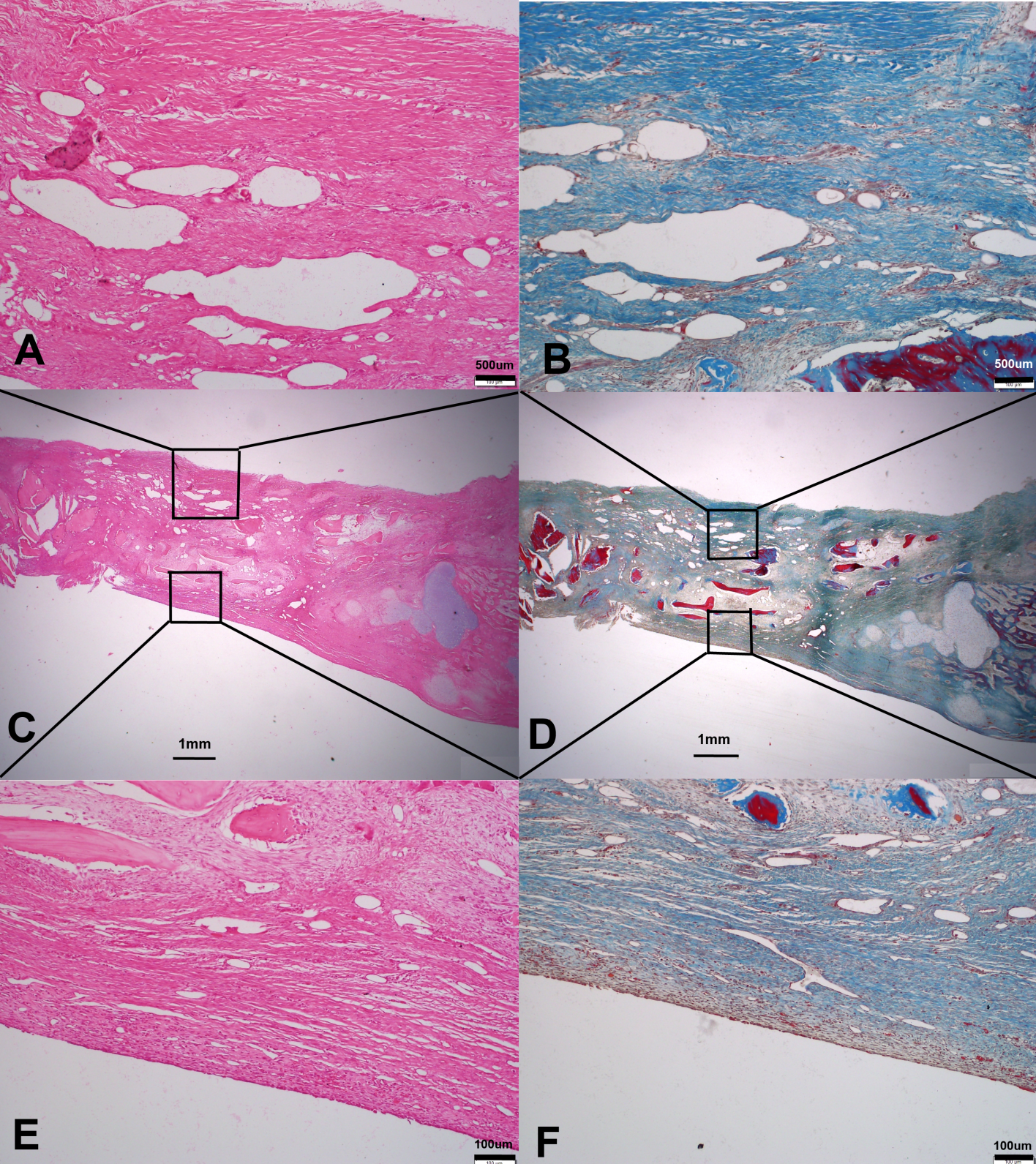

Supplement: Figure S2 — 6 week section. A representative sample of H & E and tetrachrome staining of a defect of (animal number 2386), showing the integration of the induced fibrous tissue membrane into the surrounding connective tissue by the 6 week time point. The bottom row shows the fibrous tissue orientation adjacent the stainless steel nail being slightly more directional than that of the top row where the fibrous tissue randomly blends with the surrounding tissues at the outer margin of the callus. (TIF) [file pone.0114122.s002.tif]

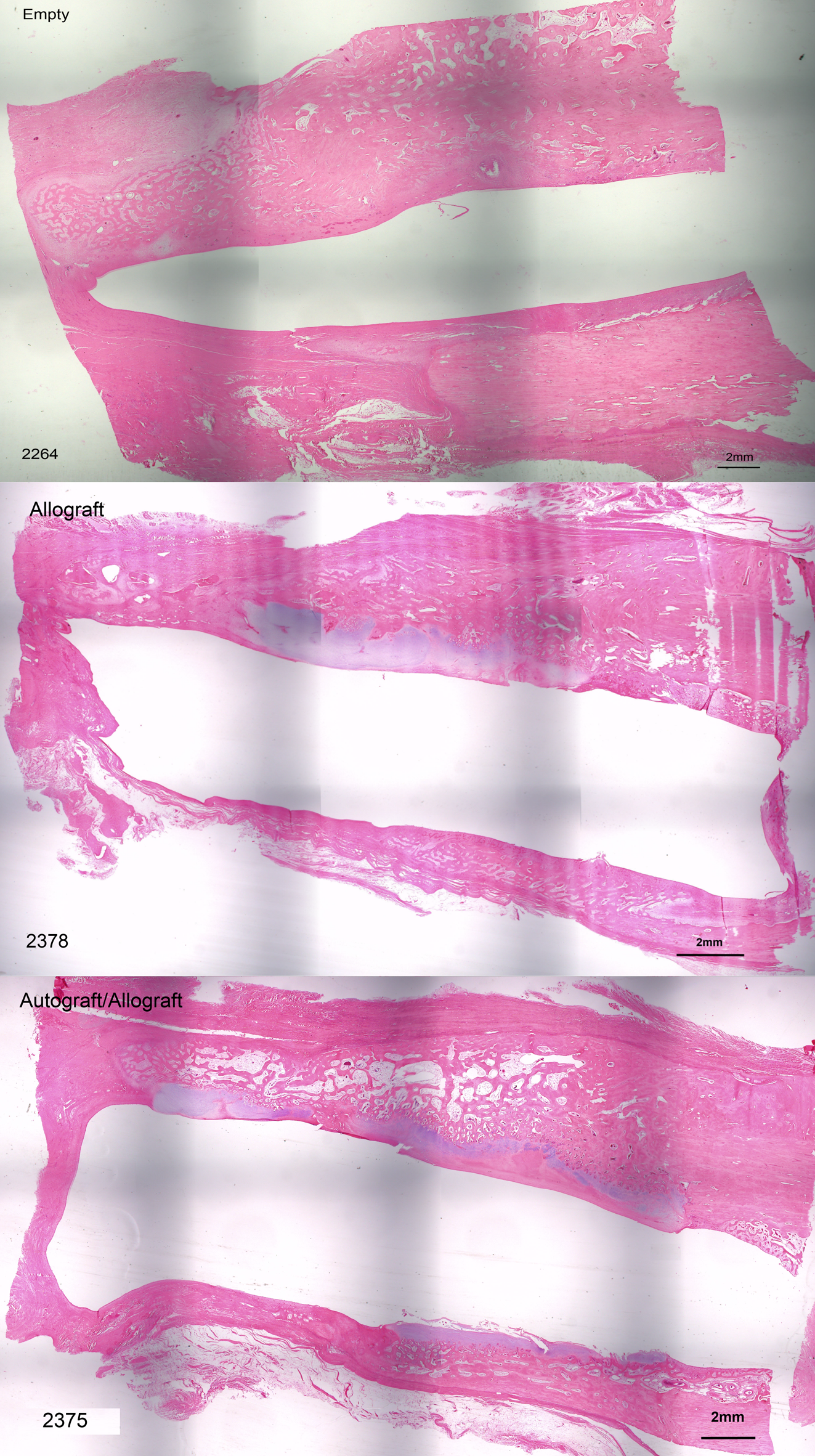

Supplement: Figure S3 — 12 week composite sections. Representative composite H & E slides of the three different groups within the study showing the amount of new bone growth from one side of the defect. (TIF) [file pone.0114122.s003.tif]

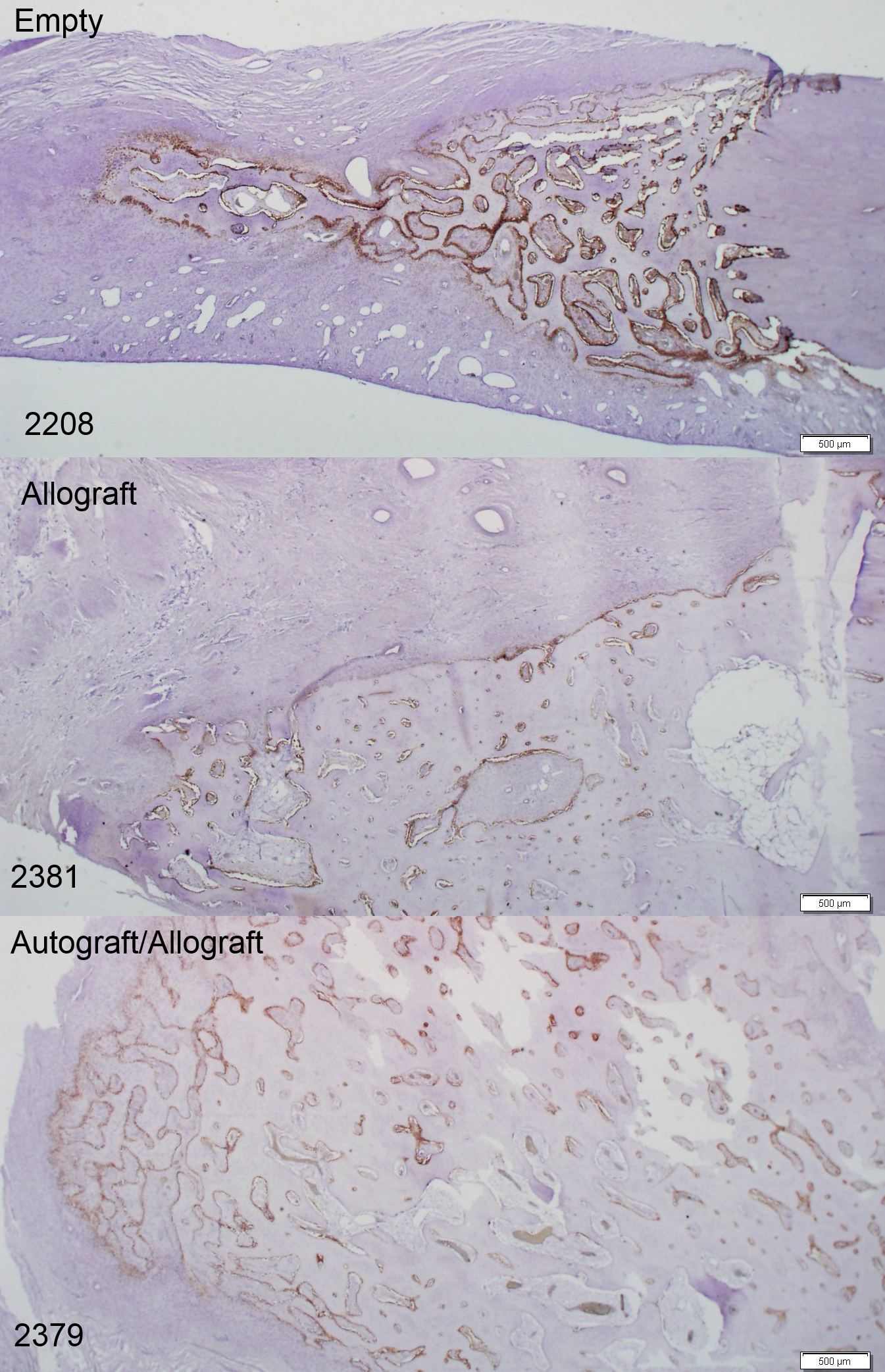

Supplement: Figure S4 — ALP expression. Representative slides of ALP staining from each of the defect groups (TIF) [file pone.0114122.s004.tif]

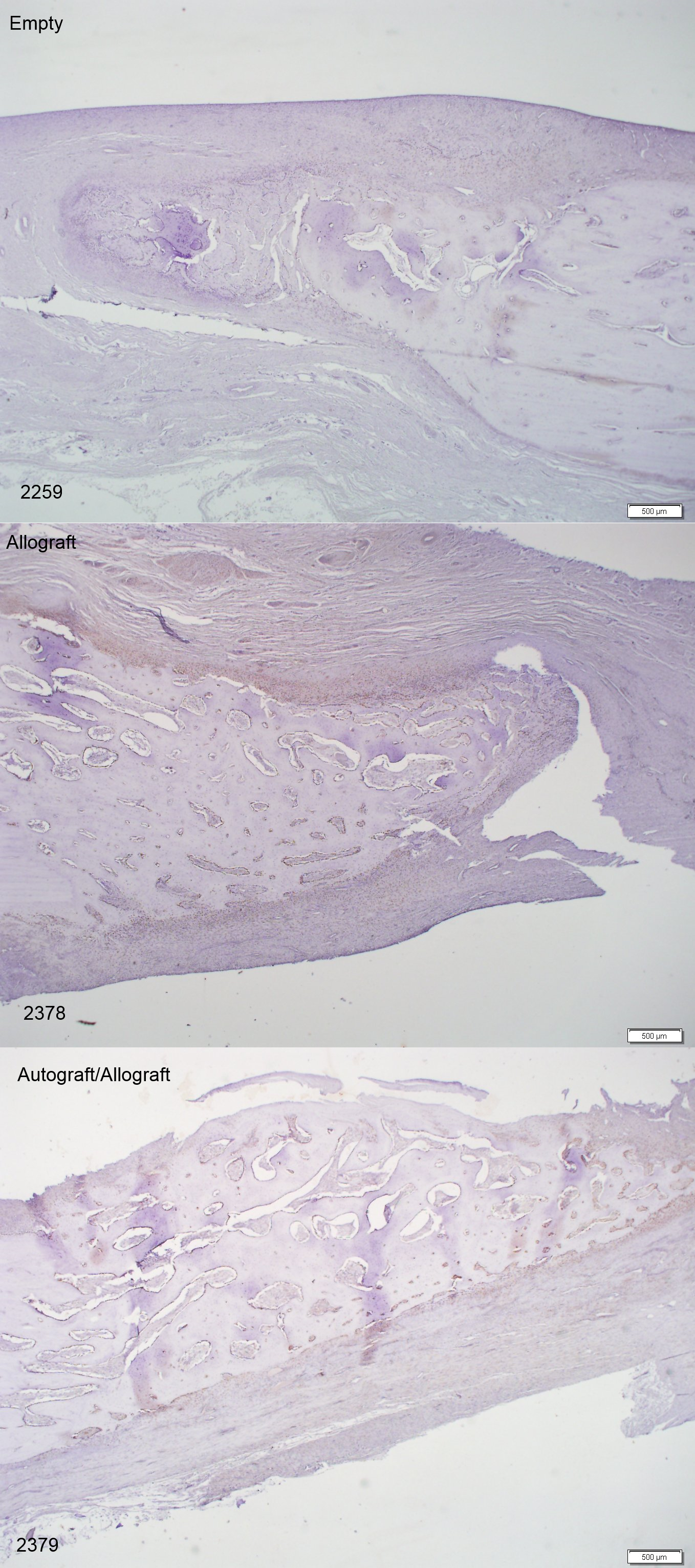

Supplement: Figure S5 — BMP2 expression. Representative slides of BMP2 staining from each of the defect groups (TIF) [file pone.0114122.s005.tif]

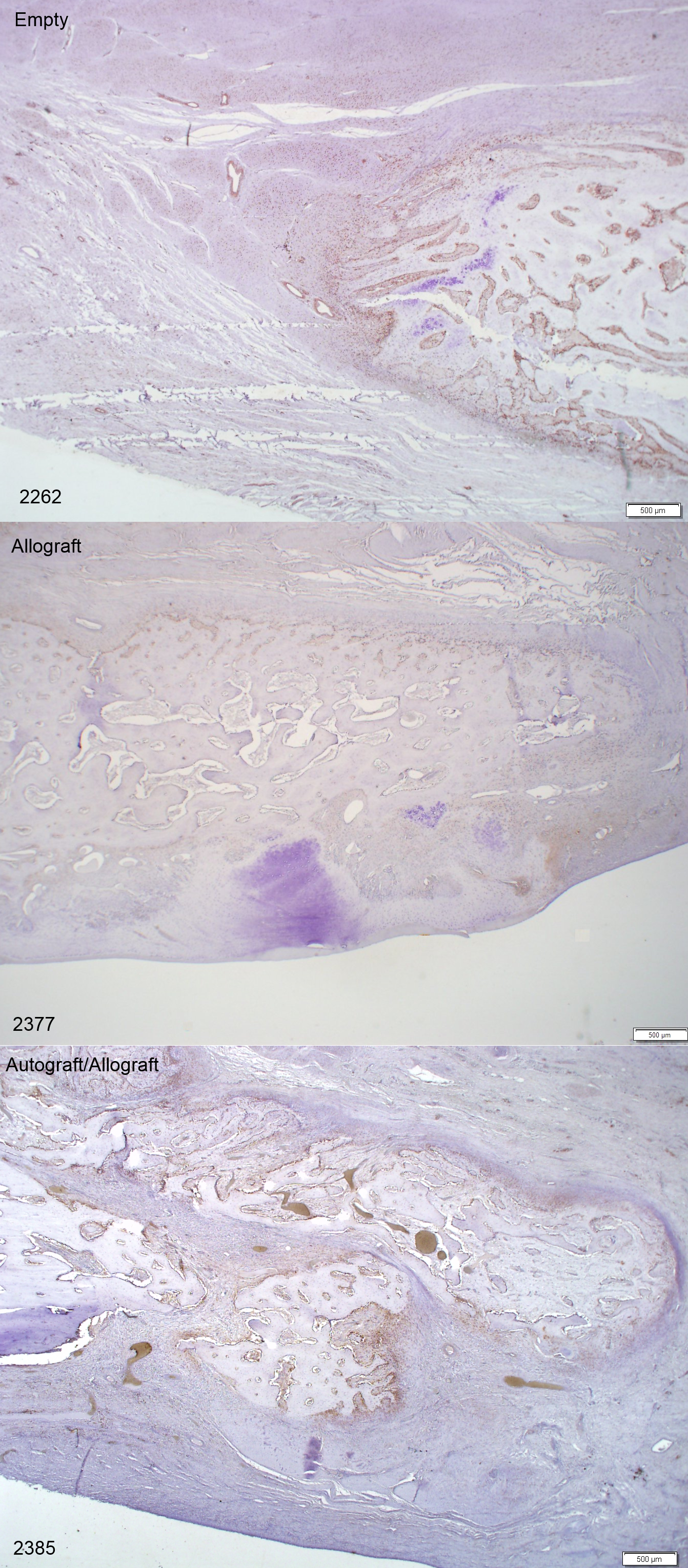

Supplement: Figure S6 — TGF-β. Representative slides of TGF-β staining from each of the defect groups (TIF) [file pone.0114122.s006.tif]

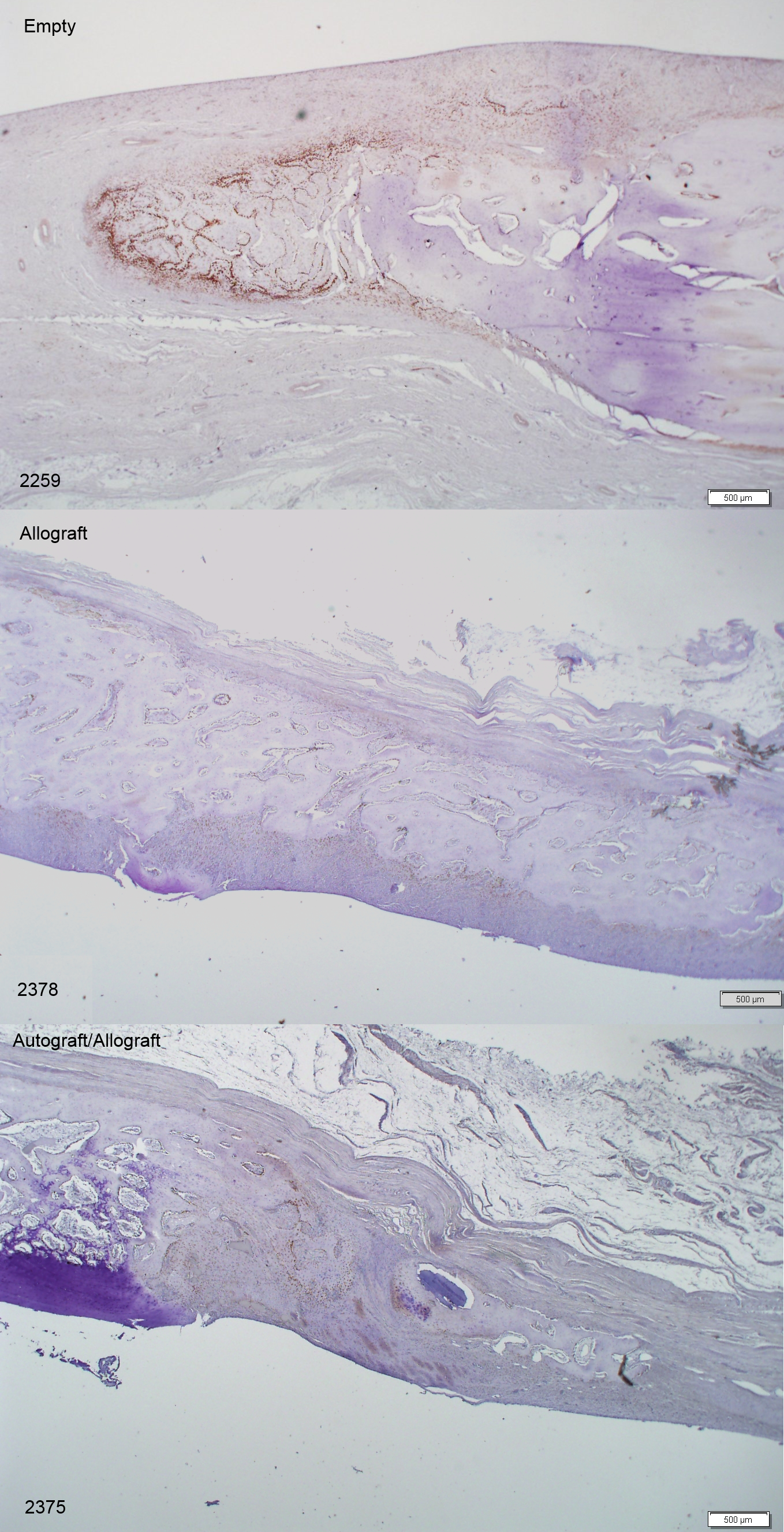

Supplement: Figure S7 — VEGF. Representative slides of VEGF staining from each of the defect groups (TIF) [file pone.0114122.s007.tif]

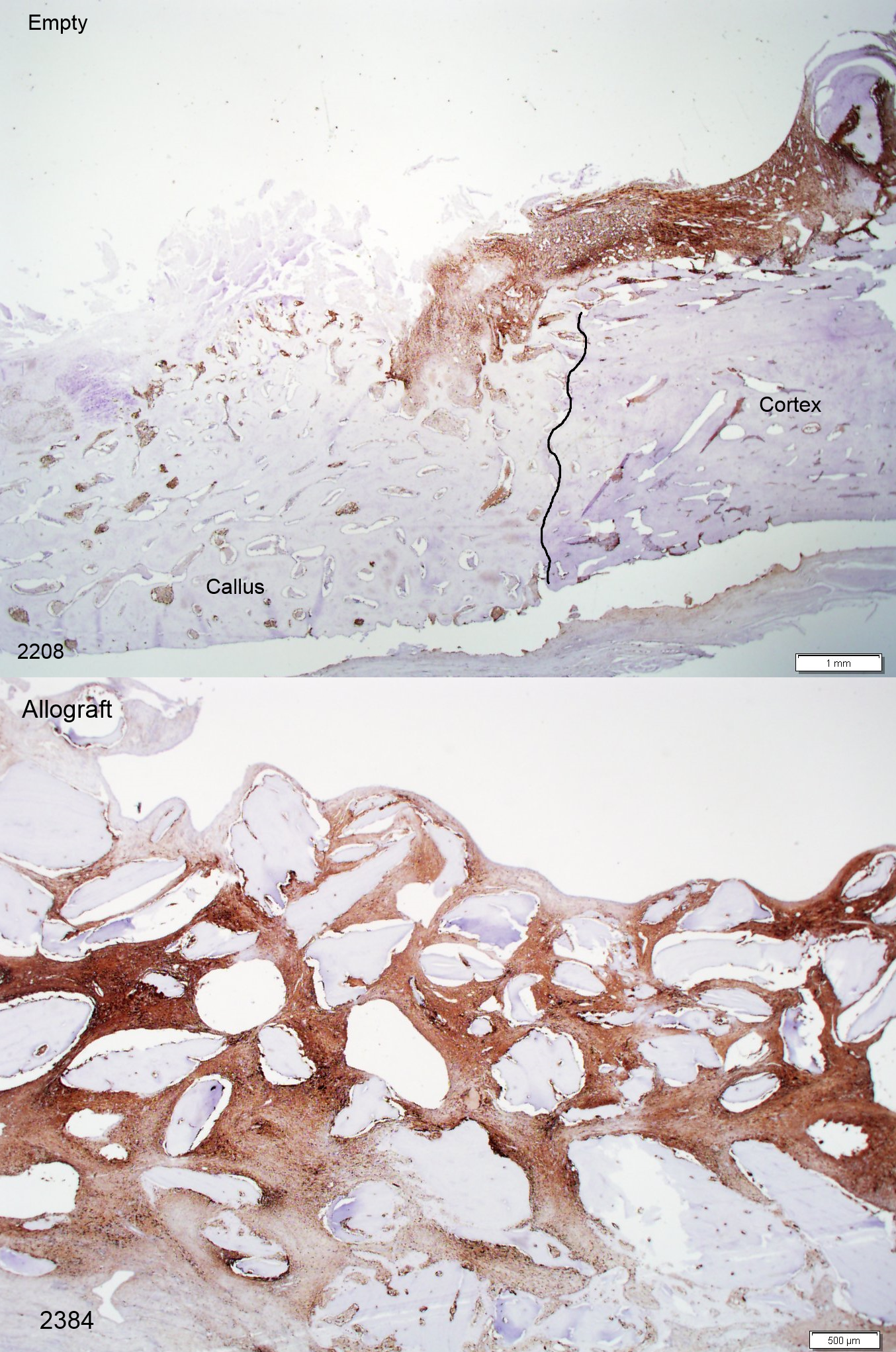

Supplement: Figure S8 — CTSK. Representative slides of CTSK staining showing activity near the osteotomy site (as indicated by the black line) top, and surrounding the graft material, below. (TIF) [file pone.0114122.s008.tif]
